# Building the case for actionable ethics in digital health research supported by artificial intelligence

**DOI:** 10.1186/s12916-019-1377-7

**Published:** 2019-07-17

**Authors:** Camille Nebeker, John Torous, Rebecca J. Bartlett Ellis

**Affiliations:** 1 Department of Family Medicine and Public Health, School of Medicine, University of California San Diego, La Jolla, CA 92093 USA; 2 Research Center for Optimal Digital Ethics in Health, Qualcomm Institute and School of Medicine, University of California San Diego, La Jolla, CA 92093 USA; 3 Beth Israel Deaconess Medical Center, Harvard Medical School, 330 Brookline Ave, Boston, MA 02215 USA; 4 School of Nursing, Indiana University, 600 Barnhill Drive, Indianapolis, IN 46202 USA

**Keywords:** Research ethics, Bioethics, Digital health, Digital medicine, Artificial intelligence, Precision medicine

## Abstract

The digital revolution is disrupting the ways in which health research is conducted, and subsequently, changing healthcare. Direct-to-consumer wellness products and mobile apps, pervasive sensor technologies and access to social network data offer exciting opportunities for researchers to passively observe and/or track patients ‘in the wild’ and 24/7. The volume of granular personal health data gathered using these technologies is unprecedented, and is increasingly leveraged to inform personalized health promotion and disease treatment interventions. The use of artificial intelligence in the health sector is also increasing. Although rich with potential, the digital health ecosystem presents new ethical challenges for those making decisions about the selection, testing, implementation and evaluation of technologies for use in healthcare. As the ‘Wild West’ of digital health research unfolds, it is important to recognize who is involved, and identify how each party can and should take responsibility to advance the ethical practices of this work. While not a comprehensive review, we describe the landscape, identify gaps to be addressed, and offer recommendations as to how stakeholders can and should take responsibility to advance socially responsible digital health research.

## Background

The digital revolution is disrupting the ways in which health research is conducted, and subsequently, changing healthcare [1–3]. The rise of digital health technologies has resulted in vast quantities of both qualitative and quantitative ‘big data’, which contain valuable information about user interactions and transactions that may potentially benefit patients and caregivers [4]. Digital data ‘exhaust’, or the traces of everyday behaviors captured in our digital experiences, are of particular interest because they contain our natural behaviors gathered in real time. No doubt, important societal conversations are needed to shape how these sociotechnical systems influence our lives as individuals, as well as the impact on society [5]. While not a formal review, this opinion essay provides a selective overview of the rapidly changing digital health research landscape, identifies gaps, highlights several efforts that are underway to address these gaps, and concludes with recommendations as to how stakeholders can and should take responsibility to advance socially responsible digital health research.

Direct-to-consumer wellness products and mobile apps (e.g., Fitbit, Strava), wearable research tools (e.g., SenseCam, ActivPAL), and access to social network data offer exciting opportunities for individuals [6], as well as traditional health researchers [7], to passively observe and/or track individual behavior ‘in the wild’ and 24/7. The volume of granular personal health data gathered using these technologies is unprecedented, and is increasingly leveraged to inform personalized health promotion and disease treatment interventions. The use of artificial intelligence (AI) tools in the health sector is also increasing. For example, electronic health records provide training data for machine learning that inform algorithms, which can detect anomalies more accurately than trained humans – particularly in the fields of cancer, cardiology, and retinopathy [8]. The digital therapeutics sector is also seeking to expand and bring products into the healthcare system, with the goal of complementing or providing an alternative to traditional medical treatments [9]. While the digital health revolution brings transformational promise for improving healthcare, we must acknowledge our collective responsibility to recognize and prevent unintended consequences introduced by biased and opaque algorithms that could exacerbate health disparities and jeopardize public trust [10, 11]. Moreover, it is critical that the minimal requirements used to make a digital health technology available to the public are not mistaken for a product that has passed rigorous testing or demonstrated real world therapeutic value [12].

Although rich with potential, the digital health ecosystem presents new ethical challenges for those making decisions about the selection, testing, implementation and evaluation of technologies in healthcare. Researchers began to study related ethical issues over 20 years ago, when electronic health records technology was being conceptualized [13], and as new forms of pervasive information communication technologies produce data, guiding principles and standards are emerging within academic research centers [14–16] and industry sectors [17, 18]. Accepted ethical principles in health research, including respect for persons, beneficence and justice, remain relevant and must be prioritized to ensure that research participants are protected from harms. Applying these principles in practice means that: people will have the information they need to make an informed choice; risks of harm will be evaluated against potential benefits and managed; and no one group of people will bear the burden of testing new health information technologies [19]. However, ethical challenges arise from the combination of new, rapidly evolving technologies; new stakeholders (e.g. technology giants, digital therapeutic start-ups, citizen scientists); data quantity; novel computational and analytic techniques; and a lack of regulatory controls or common standards to guide this convergence in the health ecosystem.

It is of particular importance that these technologies are finding their way into both research and clinical practice without appropriate vetting. For example, we have heard that, “if the product is free, then you’re the product.” This means that our search terms, swipes, clicks and keyboard interactions produce the data that companies use to inform product improvement. These ‘big data’ are used to train algorithms to produce, for example, tailored advertisements. Consumers allow this by clicking “I Accept” to confirm their agreement with the Terms and Conditions (T&C), which are not necessarily intended to be easy to read or understand. Why does this matter? When an algorithm is used to serve up a reminder about that yellow jacket you were eyeing, or the summer vacation you mentioned to a friend the other day, it may seem ‘creepy’, but it might be nice in terms of convenience. Sometimes the AI gets it right, and other times it is not even close. For example, if you were to write something on Facebook that its proprietary AI interprets as putting you at serious risk, it may send the police to your home! Is Facebook getting it right? We do not know: Facebook has claimed that, even though its algorithm is not perfect and makes mistakes, it does not consider its actions to be ‘research’ [20]. Aside from threats to one’s privacy, we should question the process of informed consent, whether there is an objective calculation of risk of harms against potential benefits, and whether people included in the product testing phase are those most likely to benefit.

## Governance in the ‘wild west’

Those involved in the development, testing and deployment of technologies used in the digital health research sector include technology developers or ‘tool makers’, funders, researchers, research participants and journal editors. As the ‘Wild West’ of digital health research moves forward, it is important to recognize who is involved, and to identify how each party can and should take responsibility to advance the ethical practices of this work.

### Who is involved?

In the twentieth century, research was carried out by scientists and engineers affiliated with academic institutions in tightly controlled environments. Today, biomedical and behavioral research is still carried out by trained academic researchers; however, they are now joined by technology giants, startup companies, non-profit organizations, and everyday citizens (e.g. do-it-yourself, quantified self). The biomedical research sector is now very different, but the lines are also blurred because the kind of product research carried out by the technology industry has, historically, not had to follow the same rules to protect research participants. As a result, there is potential for elevated risks of harm. Moreover, how and whether research is carried out to assess a product’s effectiveness is variable in terms of standards and methods, and, when the technology has health implications, standards become critically important. In addition, not all persons who initiate research are regulated or professionally trained to design studies. Specific to regulations, academic research environments require the involvement of an ethics board (known as an institutional review board [IRB] in the USA, and a research ethics committee [REC] in the UK and European Union). The IRB review is a federal mandate for entities that receive US federal funding to conduct health research. The ethics review is a peer review process to evaluate proposed research, and identify and reduce potential risks that research participants may experience. Having an objective peer review process is not a requirement for technology giants, startup companies or by those who identify with the citizen science community [10, 21]; however, we have a societal responsibility to get this right.

### What questions should be asked?

When using digital health technologies, a first step is to ask whether the tools, be they apps or sensors or AI applied to large data sets, have demonstrated value with respect to outcomes. Are they clinically effective? Do they measure what they purport to measure (validity) consistently (reliability)? For example, a recent review of the predictive validity of models for suicide attempts and death found that most are currently less than 1%; a number at which they are not yet deemed to be clinical viable [22]. Will these innovations also improve access to those at highest risk of health disparities? To answer these questions, it is critical that all involved in the digital health ecosystem do their part to ensure the technologies are designed and scientifically tested in keeping with accepted ethical principles; be considerate of privacy, effectiveness, accessibility, utility; and have sound data management practices. However, government agencies, professional associations, technology developers, academic researchers, technology startups, public organizations and municipalities may be unaware of what questions to ask, including how to evaluate new technologies. In addition, not all tools being used in the digital health ecosystem undergo rigorous testing, which places the public at risk of being exposed to untested and potentially flawed technologies.

Demonstrating value must be a precursor to the use of any technologies that claim to improve clinical treatment or population health. Value is based on the product being valid and reliable, which means that scientific research is needed before a product is deployed within the health sector [12]. We should also not move ahead assuming that privacy and the technology revolution are mutually exclusive. We are in a precarious position in which, without standards to shape acceptable and ethical practices, we collectively run the risk of harming those who stand to benefit most from digital health tools.

### Decision-making framework

**Fig. 1:**
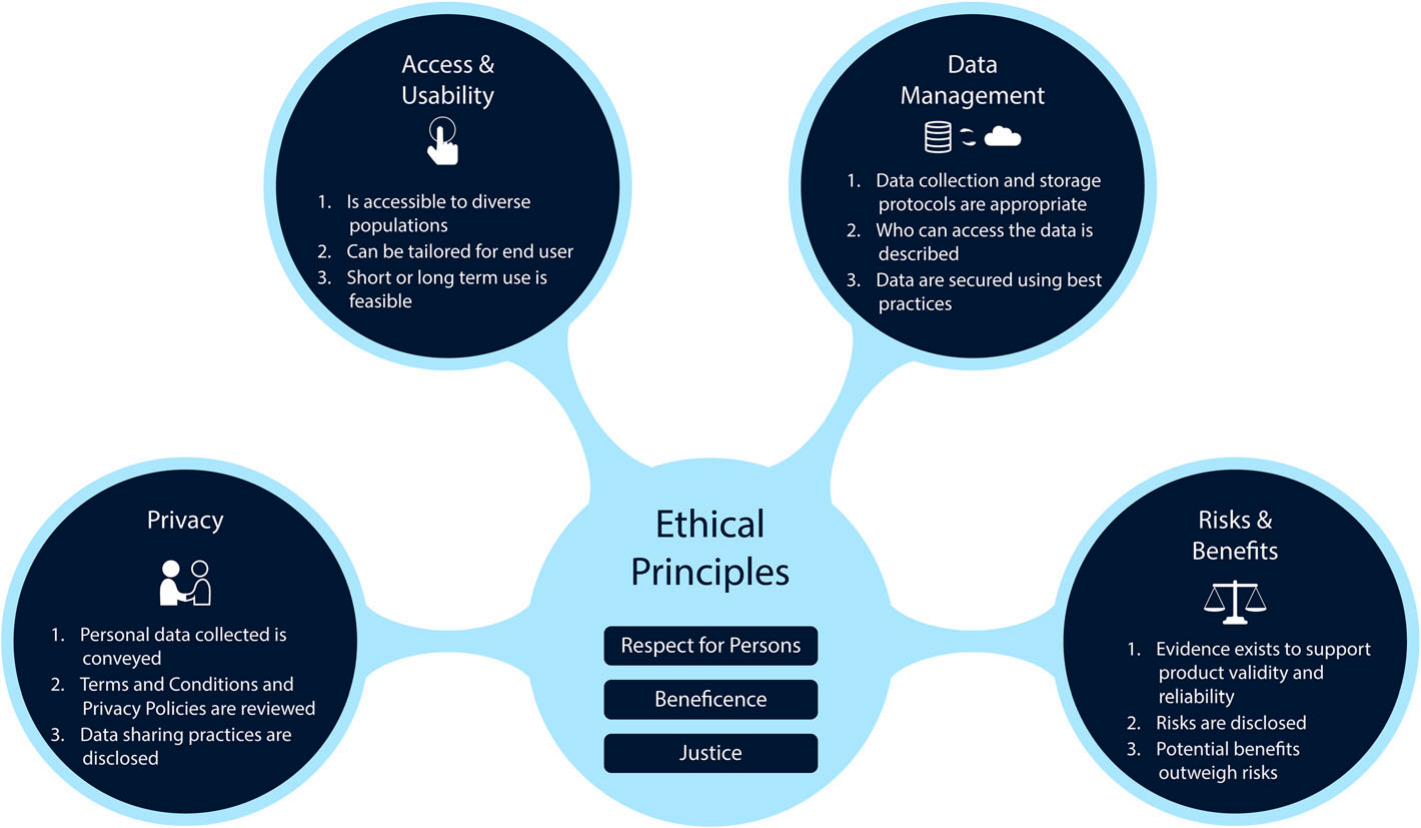
Digital health decision-making framework and excerpts from the companion checklist designed to support researchers [24]

While there are discussions about the need for regulations and laws, and incremental progress being made on that front, until some consensus is reached, it is essential that stakeholders recognize their obligation to promote the integrity of digital health research [23]. The digital health decision-making domains framework (Fig. 1) was developed to help researchers make sound decisions when selecting digital technologies for use in health research [24, 25]. While originally developed for researchers, this framework is applicable to various stakeholders who might evaluate and select digital technologies for use in health research and healthcare. The framework comprises five domains: 1, Participant Privacy; 2 Risks and Benefits; 3, Access and Usability; 4, Data Management; and 5, Ethical Principles. These five domains are presented as intersecting relationships.

The domains in this framework were developed into a checklist tool to further facilitate decision-making. The checklist was informed via developmental research involving a focus group discussion, and a design exercise with behavioral scientists [25]. To demonstrate how the decision-making domains can be put into practice, we present a use case to illustrate the complexities and nuances that are important for stakeholders to consider.

## Use case: MoodFlex for mental health

MoodFlex is a private startup technology company that has developed a mobile app to detect signals of poor mental health by analyzing a person’s typing and voice patterns from their smartphones. MoodFlex is negotiating with several municipalities to integrate their product within the public mental healthcare system, with the goal of delivering better services to people with mental illness through predictive analytics. Since MoodFlex does not claim to provide a clinical diagnosis or treatment, approval from the US Food and Drug Administration is not necessary. The vendor claims to have a proven product; however, there are no publications documenting evidence that it is safe, valid or reliable. The only research that is formally acknowledged involves an evaluation of the implementation process and uptake of the product by health providers within the state mental health system. The patient will be invited to download the app after reviewing the vendor’s T&C – no other consent process is proposed. The algorithm is proprietary, and therefore, an external body is unable to determine whether the algorithm that resulted from a machine-learning process was trained on representative data, or how decision-making occurs. Data captured about people using the app are owned by the vendor.

### Brief analysis

Before introducing MoodFlex into the public healthcare system, decision makers – particularly the funding organization – should evaluate evidence supporting the efficacy of this product. Reproducible evidence is the hallmark of evidence-based practice, and is the first step prior to dissemination and implementation. If a product is supported by evidence, the logical next step is the translational phase, in which a ‘dissemination and implementation’ (D&I) design is appropriate. Unfortunately, many health apps move straight into a D&I phase before the evidence exists to support that direction.

Lacking evidence that the product is effective, decision-makers should recognize that a testing phase is necessary. As with regulated research involving people, a research plan should be developed and reviewed by an external and objective ethics board (i.e., REC or IRB) that will assess the degree to which people who are invited do not bear an inappropriate burden (justice), potential risks are offset by the benefits (beneficence), and individuals are provided with an ability to make an informed choice to volunteer (respect). At this early stage, it is reasonable for the vendor to provide the sponsor with a robust data management plan, with explicit language regarding data ownership, access, sharing and monitoring. When involving vulnerable populations, such as those with a mental health diagnosis, additional precautions should be considered to ensure that those involved in the study are protected from harms – including stigma, economic and legal implications. In addition, it is important to consider whether some people will be excluded because of access barriers. For example, it may be necessary to adapt the technology to be useful to non-English speakers. Informed consent must also be obtained in a way that results in a person making a choice to participate based on having adequate and accessible information – this demonstrates the principle of ‘respect for persons’, and is a hallmark of research ethics. Placing consent language for a research study in the T&C is unacceptable. For patients who become research participants, it is particularly important for them to understand the extent to which the technology will support their healthcare needs. Patients might falsely rely on the technology to provide the care they believe they need when, in reality, they may need to see their healthcare provider.

## Digital research gaps and opportunities

This use case reflects the shift in health research associated with digital technologies, in that traditional methods of developing an evidence base may be pushed aside in favor of what appears to be exciting innovation. The landscape is unsettled and potentially dangerous, which makes governance important. We have identified three notable gaps: 1, disciplinary/sector challenges; 2, issues of data and technology literacy; and 3, inconsistent or non-extant standards to guide the use of AI and other emerging technologies in the healthcare settings.

### Inter/trans/cross-disciplinary and sector challenges

Emerging technologies and AI systems require diverse expertise when applied to digital medicine, which introduces new challenges. Technology makers may not understand patients’ needs, and develop tools with limited utility in practice [25, 26]. Computational scientists may train AI using datasets that are not representative of the public, limiting the ability to provide meaningful assessments or predictions [27]. Clinicians may not know how to manage the depth of granular data, nor be confident in decisions produced by AI [28]. Research is needed to examine this disconnect, and identify strategies to reduce gaps and improve meaningful connections between these groups that are integral to digital health research and the use of AI in the health care sector.

### Digital/tech-literacy

The idea that keystrokes and voice patterns can be used to aid diagnosis of Parkinson’s disease remains impressive, but now it may also be possible to use keystroke dynamics, kinematics and voice patterns to detect mental health problems [29]. Knowing this information may create public concern if not communicated in a way that is useful and contextual, adding to fear, skepticism and mistrust. The ‘public’ includes policy-makers, educators, regulators, science communicators, and those in our healthcare system, including clinicians, patients, and caregivers. Research is needed to increase our understanding of what these stakeholders know, what they want to know, and how best to increase their technology literacy. This information can then be used to inform educational resources targeting specific stakeholders. For example, when reviewing manuscripts reporting digital health research, reviewers and editors should be aware of how to evaluate new methodologies and computational analytics to verify the accuracy and appropriateness of the research and results.

### Ethical and regulatory standards

As new digital tools and AI-enabled technologies are developed for the healthcare market, they will need to be tested with people. As with any research involving human participants, the ethics review process is critical. Yet, our regulatory bodies (e.g., IRB) may not have the experience or knowledge needed to conduct a risk assessment to evaluate the probability or magnitude of potential harms [30]. Technologists and data scientists who are making the tools and training the algorithms may not have received ethics education as part of their formal training, which may lead to a lack of awareness regarding privacy concerns, risks assessment, usability, and societal impact. They may also not be familiar with regulatory requirements to protect research participants [23]. Similarly, the training data used to inform the algorithm development are often not considered to qualify as human subjects research, which – even in a regulated environment – makes a prospective review for safety potentially unavailable.

## New initiatives – what resources are available for the digital health/medicine community?

Several initiatives have begun to address the ethical, legal and social implications (ELSI) of the digital revolution in healthcare. Prominent examples of such initiatives concern AI. Specific to AI, the foci are broad, and include autonomous vehicles, facial recognition, city planning, the future of work, and in some cases, health. A few selected examples of current AI efforts appear to be well-funded and collaborative programs (see Table 1).

**Table 1:** AI initiatives underway to inform broad cross-sector standards

Program	Goal	Collaborators
The Partnership on AI [30]	Develop/test and share best practices	80+ partners in 13 countries
AI-100 [31]	Impact of AI on urban life by 2030 in North America	E. Horvitz, R. Altman
Ethics and Governance of AI Fund [32]	Conduct evidence-based research	Berkman Klein Center, Harvard and MIT Media Lab
AI Now Institute [33]	Conduct evidence-based research	New York University
Initiative on Ethics of Autonomous and Intelligent Systems [34]	Develop standards, certifications, codes	IEEE and ACM
Human Rights, Big Data and Technology Project [35]	Analyze the use of big data, artificial intelligence, associated technologies	University of Essex, United Nations
The Institute for Ethics in Artificial Intelligence [36]	Explore fundamental issues affecting the use and impact of AI	Technical University of Munich partnership with Facebook
High-Level Expert Group on Artificial Intelligence [37]	Recommend ELSI policy development on AI	European Commission
Chinese Association for Artificial Intelligence [38]	Unite artificial intelligence science and technology professionals	Ministry of Civil Affairs, China
AI for Humanity [39]	Create an international group of AI experts to prepare for societal transformation	Future of Life Institute, France

Across these initiatives are efforts to assess the potential ELSI of AI. Similar to the impact of the European Union (EU)‘s General Data Protection Regulation (GDPR) in countries beyond the EU, the intention of groups assessing AI through an ELSI lens is to develop standards that can be applied or adapted globally. In practice, however, most current efforts to integrate ELSI to AI are quite broad, and as a result, may overlap in scope and lack specificity.

While AI has a place in the digital health revolutions, the scope of technologies goes well beyond AI. Other initiatives are looking more specifically at ELSI in mobile apps, social network platforms, and wearable sensors being used in digital research. These include, for example, the Connected and Open Research Ethics (CORE) initiative at the University of California (UC) San Diego Research Center for Optimal Digital Ethics in Health (ReCODE Health), the Pervasive Data Ethics for Computational Research (PERVADE) program at the University of Maryland, and the Mobile Health ELSI (mHealthELSI) project out of Sage Bionetworks and the University of Louisville. What these initiatives have in common is a goal to inform policy and governance in a largely unregulated space. These initiatives are but a few examples, and it is important to note that many laboratories and institutes are working on digital health ELSI.

## Conclusion

Being mindful of new health technologies with new actors in the arena, the gap between known and unknown risks fundamentally challenges the degree to which decision-makers can properly evaluate the probability and magnitude of potential harms against benefits. Now is the time to take a step back and develop the infrastructure necessary for vetting new digital health technologies, including AI, before deploying them into our healthcare system. Selecting and implementing technologies in the digital health ecosystem requires consideration of ethical principles, risks and benefits, privacy, access and usability, and data management. New technologies have the potential to add important value; however, without careful vetting, may exacerbate health disparities among those most vulnerable.

## Data Availability

Not applicable.

## Abbreviations

AI: Artificial intelligence
ELSI: Ethical, legal and social implications
IRB: Institutional review board
REC: Research ethics committee
